# Probiotic Lactobacillus and Bifidobacterium Strains Counteract Adherent-Invasive *Escherichia coli* (AIEC) Virulence and Hamper IL-23/Th17 Axis in Ulcerative Colitis, but Not in Crohn’s Disease

**DOI:** 10.3390/cells9081824

**Published:** 2020-08-01

**Authors:** Gabriella Leccese, Alessia Bibi, Stefano Mazza, Federica Facciotti, Flavio Caprioli, Paolo Landini, Moira Paroni

**Affiliations:** 1 Department of Biosciences, Università degli Studi di Milano, 20133 Milan, Italy; gabriella.leccese@unimi.it (G.L.); alessia.bibi@unimi.it (A.B.); paolo.landini@unimi.it (P.L.); 2 Gastroenterology and Endoscopy Unit, Fondazione IRCCS Ca’ Granda, Ospedale Maggiore Policlinico, 20122 Milan, Italy; stefano.mazza@unimi.it (S.M.); flavio.caprioli@unimi.it (F.C.); 3 Department of Experimental Oncology, IEO European Institute of Oncology IRCCS, 20139 Milan, Italy; Federica.facciotti@ieo.it; 4 Department of Pathophysiology and Transplantation, Università degli Studi di Milano, 20135 Milan, Italy

**Keywords:** probiotics, AIEC, intestinal inflammation, IL-23/Th17, Crohn’s disease, ulcerative colitis, pro- and anti-inflammatory mechanisms

## Abstract

Hypersecretion of proinflammatory cytokines and dysregulated activation of the IL-23/Th17 axis in response to intestinal microbiota dysbiosis are key factors in the pathogenesis of inflammatory bowel diseases (IBD). In this work, we studied how *Lactobacillus* and *Bifidobacterium* strains affect AIEC-LF82 virulence mechanisms and the consequent inflammatory response linked to the CCR6–CCL20 and IL-23/Th17 axes in Crohn’s disease (CD) and ulcerative colitis (UC) patients. All *Lactobacillus* and *Bifidobacterium* strains significantly reduced the LF82 adhesion and persistence within HT29 intestinal epithelial cells, inhibiting IL-8 secretion while not affecting the CCR6–CCL20 axis. Moreover, they significantly reduced LF82 survival within macrophages and dendritic cells, reducing the secretion of polarizing cytokines related to the IL-23/Th17 axis, both in healthy donors (HD) and UC patients. In CD patients, however, only *B. breve* Bbr8 strain was able to slightly reduce the LF82 persistence within dendritic cells, thus hampering the IL-23/Th17 axis. In addition, probiotic strains were able to modulate the AIEC-induced inflammation in HD, reducing TNF-α and increasing IL-10 secretion by macrophages, but failed to do so in IBD patients. Interestingly, the probiotic strains studied in this work were all able to interfere with the IL-23/Th17 axis in UC patients, but not in CD patients. The different interaction mechanisms of probiotic strains with innate immune cells from UC and CD patients compared to HD suggest that testing on CD-derived immune cells may be pivotal for the identification of novel probiotic strains that could be effective also for CD patients.

## 1. Introduction

The two major types of inflammatory bowel diseases (IBD) include Crohn’s disease (CD) and ulcerative colitis (UC). The former is characterized by chronic inflammation involving most commonly the distal part of the small intestine, ileum and colon, while the latter is characterized by a more restricted inflammation localized to the colonic mucosa and submucosa [1]. IBD pathogenesis is a complex process, mainly linked to uncontrolled mucosal inflammation in genetically predisposed individuals, due to an abnormal immune response against luminal antigens and microbiota [2,3,4]. This overly inflammatory response can be triggered by a breakdown in the intestinal homeostasis among the microbiota and resident innate and adaptive immune cells [5,6]. Consistent with this notion, the gut microbiota of IBD patients exhibit a reduction in total bacterial diversity [7,8,9]. Indeed, several metagenomic studies of human gut microbiota have revealed a hallmark shift, in both CD and UC patients compared to healthy individuals, from predominant “symbiont” microorganisms to potential harmful “pathobionts”, characterized by an expansion of Enterobacteriaceae at the expense of beneficial bacteria, such as *Fusobacterium, Clostridium, Ruminococcus, Lactobacillus* and *Bifidobacterium* genus [10,11,12]. Indeed, an increase in mucosa-associated *Escherichia coli* strains is characteristic of IBD patients: in particular, the adherent-invasive *E. coli* (AIEC) pathotype, highly enriched in the inflamed ileum of CD patients and present also in colonic mucosa of UC patients, has been suggested to be an important contributor to IBD pathogenesis [13,14,15]. Several virulence factors confer AIEC the ability to adhere, invade, survive and replicate within host cells, resulting in severe barrier dysfunction [16]. Consequently, AIEC triggers a strong proinflammatory response via a series of signaling pathways: AIEC strains proficiently evade clearance and replicate within phagocytic cells inducing the release of tumor necrosis factor-α (TNF-α) [17,18] and also cardinal proinflammatory polarizing cytokines which drive Th1/Th17 differentiation [19]. In fact, even if IBD appear to differ in their inflammatory mechanisms, with CD having been classically linked to Th1 and Th17 cells, while UC to an atypical Th2 and Th17 condition [20,21], the IL-23/Th17 axis is thought to play a central role in both conditions [22,23]. Strong evidence for this common mechanism comes both from the elevated expression of IL-17 and IL-23 in the gut mucosa of active UC and CD patients compared to healthy controls and from genome-wide association studies linking single nucleotide polymorphisms (SNPs) in the IL-23 receptors (IL-23R) and in STAT3 to an increased risk for IBD [24,25]. Notably, it is now widely accepted that IL-23, predominantly produced by activated dendritic cells in response to both pathogenic and nonpathogenic bacteria [26,27,28], plays a fundamental role—together with IL-1β—in the differentiation and maintenance of pathogenic Th17 cells, in turn contributing to the pathogenesis of IBD [29,30]. Therefore, inhibiting the activation of the IL-23/Th17 pathway may have a therapeutic potential in IBD [31]. Indeed, many anti-inflammatory drugs are commonly used to treat IBD, such as mesalazine (5-ASA) and 6-mercaptopurine (6MP), however, although they have proven effective in decreasing intestinal inflammation, they induce a sustained remission in only a minority of patients [32]. Moreover, they also lead to a nonspecific decrease in relative abundance of both pathogenic and beneficial commensal bacteria [33,34] thus exacerbating gut dysbiosis.

Therefore, selective blocking of the IL-23 dependent Th17 differentiation pathway through the employment of the anti-inflammatory properties of probiotic strains, simultaneously restoring the eubiotic state of the gut microbiota ecosystem, remains an attractive therapeutic strategy in IBD [35]. Many works have reported the positive effects of distinct probiotic strains in induction or maintenance of remission, both in UC and in CD patients [36,37]. Although the understanding of probiotic mechanisms of action has recently improved considerably, many questions on the functional role of distinct probiotic strains in modulating the innate immune response related to the IL-23/Th17 axis in CD and UC patients remain to be addressed.

In this study, we compared the ability of distinct probiotic strains belonging to either *Lactobacillus* and *Bifidobacterium* genus, among the most commonly present bacteria to be found in commercially available probiotic preparations used in the treatment of IBD [36,37], to affect AIEC virulence mechanisms and interfere with the relative inflammatory response directly related to the IL-23/Th17 axis. For the strain-specific nature of the immunomodulatory effects of probiotic strains, we decided to evaluate the specific ability of two *Lactobacillus* and two *Bifidobacterium* strains to modulate AIEC-LF82 invasion and survival within intestinal epithelial cells (IECs), macrophages and dendritic cells isolated from HD and IBD patients. Finally, we tested their potential to impair the CCL20-dependent and IL-23-dependent Th17 cells recruitment and differentiation pathways, in comparison to the anti-inflammatory drug 6-mercaptopurine (6MP).

Our data displayed, for the first time, that *Lactobacillus* and *Bifidobacterium* probiotic strains differentially affect AIEC virulence based on immune cell origin and show different immunomodulatory effects on the IL-23/Th17 axis in UC and CD patients.

## 2. Materials and Methods

### 2.1. Bacterial Strains and Culture Conditions

Bacterial strains used in this study are listed in Table 1. *Bifidobacterium* strains were grown in MRS medium (BD Difco™, Franklin Lakes, NJ, USA) supplemented with a 0.25% L-cysteine (Sigma Chemical Co, Darmstadt, Germany) and incubated at 37 °C in anaerobic conditions for 24 h. *Lactobacillus* strains were cultured in LB medium (BD Difco™) supplemented with a 2% L-lactose (Sigma Chemical Co) under static conditions at 37 °C for 24 h. *Staphylococcus epidermidis* was grown under aerobic conditions, in YESCA medium (10 g/L casamino acids, 1.5 g/L yeast extract) at 37 °C for 24 h. AIEC-LF82 strain was grown under aerobic conditions, in YESCA medium supplemented or not with 2 µg/mL of 6-mercaptopurine (6MP) [38], at 37 °C for 18 h. In order to determine bacterial titer (CFU/mL) for infection/coinfection experiments, bacterial cultures were centrifuged and, after spectrophotometer quantification (Ultrospec 3000—Pharmacia Biotech Cambridge, UK), resuspended to an OD_600_ = 1. Serial dilutions were plated onto either MRS agar (*Bifidobacterium* strains) or LB agar supplemented with 2% lactose (*Lactobacillus* strains) or LB agar (LF82, *S. epidermidis*). Plates were incubated at 37 °C for 24–48 h.

### 2.2. Human Peripheral Blood

Human peripheral blood was collected either from HD with no history of immune-mediated diseases, allergies or malignancies (*n* = 10) or from patients with active UC (*n* = 11) or CD (*n* = 8) following informed consent.

All IBD patients included in this study were not receiving immunosuppressive therapies and/or antibiotics and were selected according to the disease activity, using Harvey–Bradshaw index (HBI) for CD patients or Mayo score for UC patients as previously described [40]. The clinical characteristics of the included patients and healthy subjects are listed in Supplementary Table S1. The study was approved by the local ethics committee (Milano Area B), code 566_2015, and was performed in accordance with the Declaration of Helsinki protocols.

### 2.3. HT29 Cell Line Culture Conditions and Infection Assays

HT29 cells were seeded at 4 × 10^5^ cells per well into 24-well tissue culture plates and grown to confluence (4 days) in complete RPMI medium (10% heat-inactivated FCS, 1 mmol/L sodium pyruvate, 10 mmol/L non-essential amino acids and 1% penicillin/streptomycin). Each confluent monolayer was infected with LF82 alone or in combination with *Bifidobacterium* strains (B_1_ or B_2_) or *Lactobacillus* strains (L_1_ or L_2_) or *S. epidermidis*, grown in different conditions and resuspended in complete RPMI without penicillin/streptomycin, at the final concentration of 7 × 10^6^ CFU/mL. For coinfection experiments, AIEC-LF82 was mixed at a 1:1 ratio with each probiotic strains or with *S. epidermidis*. For the experiments with the anti-inflammatory drug 6MP, LF82 was pre-grown in YESCA supplemented with 2 µg/mL 6MP (a subinhibitory concentration for LF82 growth inhibition) [38] and the same concentration was added to confluent monolayers in complete RPMI without penicillin/streptomycin and maintained throughout the course of infection (referred to as +6MP in all figure legends). After 3 h and 7 h incubation periods, adhesion and invasion of LF82 was measured using the gentamicin protection assay as previously described [38]. All bacteria strains were highly sensitive to gentamicin. Results are expressed as cell-associated (adherent + intracellular) or intracellular LF82 cells in coinfections conditions relative to those obtained for LF82 alone, set to 100%.

### 2.4. Biofilm Formation

Biofilm formation of LF82 either alone or in combination with *Bifidobacterium* strains (B_1_ or B_2_) or *Lactobacillus* strains (L_1_ or L_2_) or *S. epidermidis*, was determined using the crystal violet (CV) assay. Bacterial cultures, grown in different conditions, were diluted to OD_600_ = 0.02 and then incubated in triplicates (200 µL/well) in a polystyrene 96-well round bottom plate for 16 h at 37 °C. For coinfection experiments, LF82 and each of the other bacterial strains were mixed (ratio 1:1) and incubated in triplicates as before. LF82 was also grown in the presence of 2 µg/mL 6MP which strongly inhibits biofilm formation. After 18 h of incubation, the plates were washed with phosphate-buffered saline (PBS) to remove unattached strains and then stained with 1% CV solution for 20 min at room temperature. After washing, adherent stains were dissolved in a solution containing 96% ethanol in water and quantified at 550 nm in a microplate reader (SAFAS, MP96, Monaco). The adhesion index of LF82, alone or in competition experiments, was calculated as OD_550_(CV)/OD_600_ (planktonic culture). The adhesion index of LF82 alone was set as 100%, and results are expressed as adhesion index of LF82 in coinfections relative to adhesion index of LF82 alone.

### 2.5. Generation of Monocyte-Derived Macrophages (MDM) and Monocyte-Derived Dendritic Cells (MoDC)

Human monocytes were purified from heparinized blood samples derived from CD, UC or healthy subjects by Ficoll density gradient separation and by positive selection using CD14^+^ selection (CD14 Microbeads, Miltenyi Biotec, Bergisch Gladbach; Germany).

For generation of monocyte-derived macrophages (MDM), CD14^+^ cells were seeded into flat bottom 96-well culture plates at a density of 2 × 10^5^ cells/well in complete RPMI medium supplemented with 100 ng/mL of recombinant human macrophage-colony stimulating factor (rh-M-CSF, Miltenyi Biotec). The cells were incubated in a humidified atmosphere at 37 °C with 5% CO_2_. After 2 and 4 days, all the unattached cells were discarded by removing half of the culture media and replacing it with complete RPMI with twice the final concentration of M-CSF. On day 7 the total number of MDM cells per well, as well as the morphology and the expected immunophenotype, were confirmed by flow cytometry (data not shown).

For generation of monocyte-derived dendritic cells (MoDC), CD14^+^ cells were cultured into 24-well culture plates at a density of 10^6^ cells/mL in complete RPMI medium supplemented with 100 ng/mL of recombinant human granulocyte–monocyte colony stimulating factor (rh-GM-CSF, Miltenyi Biotec) and 50 ng/mL of Interleukin-4 (IL-4, Miltenyi Biotec). After 3 days, culture media were replaced by removing half of the complete medium and replacing it with complete RPMI with twice the final concentration of cytokines used for differentiation. Thereafter, MoDC were collected and seeded into round bottom 96-well culture plates at a density of 10^5^ cells/well in complete RPMI medium without pen/strep for the coinfection experiments. The morphology, as well as the immunophenotype analysis to check for proper differentiation of monocytes into MoDC, were confirmed at this time point by flow cytometry before the infection experiments (data not shown).

### 2.6. Phagocytosis and Intracellular Survival Assays

MDM monolayers and MoDC were infected with 10^6^ CFU/mL of LF82 alone (MOI 10:1 bacteria per phagocytic cells) or coinfected with LF82 together with each *Bifidobacterium* strains (B_1_ or B_2_) or single *Lactobacillus* strains (L_1_ or L_2_) or *S. epidermidis* mixed in a 1:1 ratio (final concentration of 10^6^ CFU/mL for each strain). Following 1 h of phagocytosis, gentamicin (20 µg/mL, Sigma-Aldrich, Darmstadt, Germany) was added to kill extracellular bacteria. Subsequently, infected MDM and MoDC were washed and maintained in RPMI supplemented with gentamicin (2 µg/mL) for additional 7 and 23 h, respectively. At each time point, MDM and MoDC were washed twice with PBS, and cells were lysed with a solution of Triton X-100 (1% *vol/vol,* Sigma-Aldrich) in deionized water for 15 min to release internalized bacteria. The number of internalized LF82 bacterial cells within MDM or MoDC, either in simple infections or in coinfections with probiotic strains, was determined by plating serial dilutions on LB agar plates, and CFU were determined after 24 h growth at 37 °C by viable count. Results were expressed as percentage of intracellular LF82 cells in coinfection experiments compared to percentage of intracellular LF82 in simple infections, taken as 100%.

### 2.7. Immunofluorescence and Confocal Microscopy

MDM monolayers on glass coverslips were infected with LF82 alone or coinfected with LF82 and *Lactobacillus* L_1_ as previously described. After 1 h of incubation in RPMI medium and 1 h of incubation with gentamicin, cells were washed, fixed in 4% paraformaldehyde/PBS, blocked in 2% FBS/PBS and stained with rabbit anti-*E. coli* antibody (ab20640, 1:100, Abcam, Cambridge, UK) for 1 h. Coverslips were then incubated with anti-rabbit Alexa Fluor 488 (1:500 Invitrogen, Eugene, Oregon, USA) for 1 h, washed, and then nuclei were stained with 4′,6-diamidino-2phenylindole (DAPI, Molecular Probes, Eugene, Oregon, USA). The Z-stacks images were acquired by a Nikon A1 laser scanning confocal microscopy using a 20× dry objective (NA 0.75) or a 60× oil immersion objective (NA 1.4).

### 2.8. Cytokine Analysis

IL-8 (IL-8/CXCL8 ImmunoTools, Friesoythe, Germany) and chemokine (C–C motif) ligand 20 (CCL20/MIP-3α, R&D Systems, Minneapolis, MN, USA) secretion by intestinal epithelial HT29 cells after 7 h of infection was analyzed by ELISA assays according to the manufacturer’s instructions.

Supernatants of MDM were collected at 8 h post-infection and analyzed for TNF-α, (BioLegend, San Diego, CA, USA), IL-6 (ImmunoTools) and IL-10 (Thermo Fisher, Rockford, IL, USA) by ELISA assays. The levels of IL-1β (BioLegend), IL-12p70 (BioLegend), IL-23p19 (R&D Systems) and IL-10 (Thermo Fisher) in supernatants of infected MoDC were analyzed after 24 h by ELISA assays. The Elisa plates were read on microplate reader (SAFAS MP96), and data were analyzed with Prism software (version 7; GraphPad Software, Inc., La Jolla, CA, USA).

### 2.9. Statistics

Independent sample groups were assessed for normality and equality of variances. Statistical significance was evaluated by nonparametric Wilcoxon signed-rank test (100% as reference value) to analyze variables that were not normally distributed or by one sample t-test to analyze variables that were normally distributed. The p-values were corrected using FDR.

For not normalized data, statistical significance was evaluated by nonparametric Kruskal–Wallis test (Dunn’s multiple comparison) to analyze variables that were not normally distributed or by one-way ANOVA (Dunnett’s multiple comparisons) to analyze variables that were normally distributed. All experiments were performed at least 3 times.

Significance was defined at *p* < 0.05. Statistical calculations were performed with Prism software (version 7; GraphPad Software, Inc., La Jolla, CA, USA).

## 3. Results

### 3.1. Probiotic Strains Reduced AIEC-LF82 Invasion of IECs, Blocking the Inflammatory Response, but Not the CCR6-CCL20 Axis

**Figure 1:**
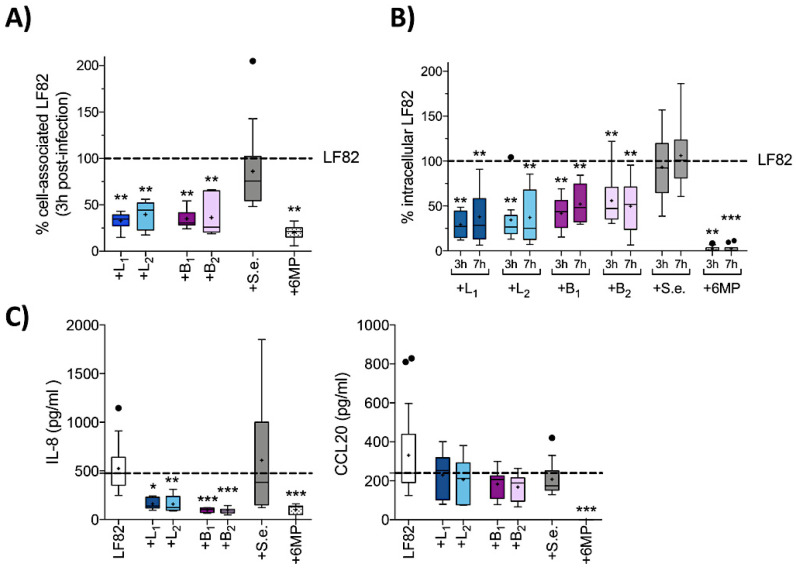
Effects of probiotic strains on adhesion and invasion of intestinal epithelial cells (IECs) by AIEC-LF82 and the relative inflammatory response. (**A**) Adhesion to HT29 cell monolayers of AIEC-LF82 alone (taken as 100%) or in the presence of *Lactobacillus acidophilus* LA1 (+L_1_), *Lactobacillus paracasei* 101/37 (+L_2_), *Bifidobacterium animalis* spp. *lactis Bi1* (+B_1_), *Bifidobacterium breve Bbr8* (+B_2_), *S. epidermidis* ATCC-155 (+S.e.) at 1:1 ratio or in the presence of 6-mercaptopurine (+6MP, 2 µg/mL) was quantified after a 3 h incubation period; (**B**) invasion of AIEC-LF82 alone (taken as 100%) or in the presence of probiotic strains or *S. epidermidis* (+S.e.) at 1:1 ratio or in the presence of 6MP was quantified after a 3 h or 7 h incubation period. Results are expressed as the percentage of cell-associated LF82 (adherent plus intracellular LF82 cells) or intracellular LF82 relative to those obtained in monoinfection with LF82 alone, taken as 100%; (**C**) IL-8 and CCL20 secretion by HT29 cells after 7 h of infection with AIEC-LF82 alone or in the presence of probiotic strains or *S. epidermidis* (+S.e.) at 1:1 ratio or in the presence of 6MP was quantified by ELISA. Dot lines (---) represent the median values for cytokine secretion of HT29 cells infected with LF82 alone. Each experiment was performed in triplicate, and data are represented with box-plots showing the median, range and upper and lower quartiles of at least three independent experiments. Means are represented as black cross (+), median as horizontal lines, and outliers are marked as dots. Statistical significance for each condition compared to HT29 infected with LF82 alone was reported (* *p* < 0.05, ** *p* < 0.01, *** *p* < 0.001).

As several probiotic strains can inhibit AIEC invasion of IECs [41,42], we first assessed whether the Lactobacillus (L_1_—*Lactobacillus acidophilus* LA1, L_2_—*Lactobacillus paracasei* 101/37) or Bifidobacterium (B_1_—*Bifidobacterium animalis* spp. *Lactis* Bi1, B_2_—*Bifidobacterium breve* Bbr8) strains were able to impair AIEC invasion of IECs and the consequent inflammatory response. HT29 cells were coinfected with the AIEC-LF82 strain either alone or in the presence of the probiotic strains at a 1:1 ratio (Figure 1). We chose these strains for our study, as they are highly representative of probiotic most widely used in IBD and are found in the commercial probiotic formulation LD Proactiv@ 50 (Named SpA, Italy). To verify that inhibition of LF82 invasion was not simply due to competition by any bacterial strain, we also performed coinfection experiments in the presence of *S. epidermidis* ATCC 155, a nonprobiotic Gram-positive strain. The anti-inflammatory drug 6MP, which is also a strong inhibitor of LF82 virulence determinants [38], was also tested in these experiments for comparative purposes.

Our results show that both *Lactobacillus* (L_1_ and L_2_) and *Bifidobacterium* (B_1_ and B_2_) strains, consistent with their probiotic nature, and in contrast to *S. epidermidis*, significantly reduced LF82 adhesion to HT29 cells, as well as biofilm formation (Supplementary Figure S1A), thus suggesting that probiotic strains may inhibit LF82 adhesion by blocking specific adhesion determinants.

Next, we assessed the ability of probiotic strains to modulate the invasion and survival of LF82 within IECs at either 3 and 7 h after infection (Figure 1B), as well as the induction of proinflammatory chemokines IL-8 and CCL20 at 7 h post-infection (Figure 1C). In the presence of either *Lactobacillus* strain, LF82 invasion (Figure 1B) was significantly impaired probably as a consequence of reduced adhesion to IECs (Figure 1A). Likewise, B_1_ and B_2_ reduced LF82 invasion and survival, albeit to a slightly lesser extent than L_1_ and L_2_. Finally, 6MP was able to completely hamper LF82 invasion and survival within HT29 cells (*p* < 0.001), while no effect was observed in coinfections with *S. epidermidis*.

IECs respond to bacterial invasion by releasing IL-8 and CCL20, a pivotal step in the recruitment of granulocytes and CCR6^+^ lymphocytes (Th17 and Th1/17 cells) to the site of infection. Consistently, in confluent HT29 cell monolayers, LF82 triggered high levels of IL-8 and CCL20 after 7 h of infection (Figure 1C), while either *S. epidermidis* or single probiotic strains induced little or no secretion of either cytokine (Supplementary Figure S1B).

Interestingly, despite lower inhibition of LF82 survival in IECs compared to the anti-inflammatory drug 6MP, probiotic bacteria induced a similar reduction of IL-8 secretion in co-culture experiments. This was observed both with *Lactobacillus* strains (from 475.3 pg/mL to 139.7 pg/mL L_1_, and to 123.7 pg/mL L_2_; respectively) and *Bifidobacterium* strains (102.1 pg/mL B_1_, 86.77 pg/mL B_2_; respectively), but not with the nonprobiotic *S. epidermidis* (383.1 pg/mL). In contrast, no suppressive effect was observed on the production of CCL20 with any bacteria tested (Figure 1C), indicating that a significant reduction in intracellular bacterial load is not sufficient to attenuate the production of CCL20 by HT29 cells and suggesting a direct effect of probiotics on IL-8 production only.

Our results suggest that probiotic strains can efficiently reduce the number of AIEC-LF82 that reach sub-epithelial regions, affecting the invasion and survival of LF82 within intestinal epithelial cells and strongly attenuate the secretion of IL-8, but not of CCL20, an important chemokine involved in the recruitment of DCs, Th17 and Th1/17 cells to the gut mucosa [43,44].

### 3.2. Probiotic Bacteria Reduced Phagocytosis and Intramacrophage Replication of AIEC-LF82 in HD and UC Patients, but Not in MDM Derived from CD Patients

**Figure 2:**
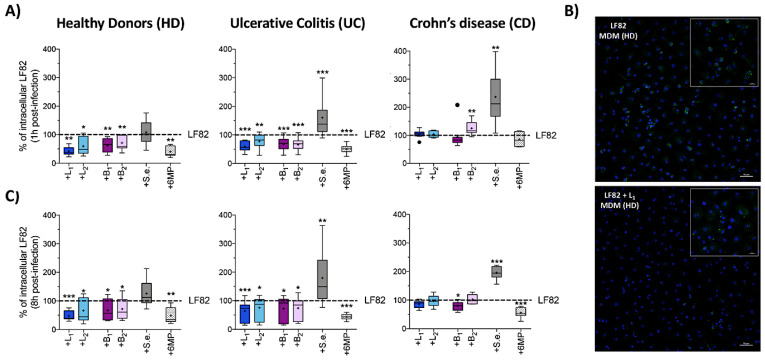
Effects of probiotic strains on phagocytosis and intracellular survival of AIEC-LF82 within human monocyte-derived macrophages (MDM). Percentage of internalized AIEC-LF82 cells within MDM derived from HD, UC or CD patients after 1 h (**A**) or 8 h of infection (**C**) with LF82 alone (taken as 100%) or in coinfection with *Lactobacillus acidophilus* LA1 (+L_1_) or *Lactobacillus paracasei* 101/37 (+L_2_) or *Bifidobacterium animalis* spp. *lactis Bi1* (+B_1_) or *Bifidobacterium breve Bbr8* (+B_2_) or *S. epidermidis* ATCC-155 (+S.e.), at 1:1 ratio or in the presence of 6MP (2 µg/mL). Each experiment was performed in triplicate and data are represented as described in Figure 1. Statistical significance for each condition compared to MDM infected with LF82 alone was reported (* *p* < 0.05, ** *p* < 0.01, *** *p* < 0.001); (**B**) confocal microscopic examination of HD-derived MDM infected with AIEC-LF82 alone or in coinfection with *L. acidophilus* LA1 (+L_1_) at 1 h post infection. Cell nuclei (stained by DAPI) are shown in blue and AIEC-LF82 in green. Larger images: magnification 200×, scale bars: 50 µm. Smaller images: magnification 630×, scale bars: 20 µm.

AIEC strains can survive and replicate within macrophages without inducing host cell death and promoting secretion of high amount of TNF-α [17,18]. In order to establish the ability of probiotic strains to reduce AIEC survival and replication within macrophages, MDM isolated from HD, UC and CD patients were coinfected with LF82 and *Lactobacillus* or *Bifidobacterium* strains in a 1:1 ratio (Figure 2); *S. epidermidis* and 6MP were included in our experiments for comparative purposes.

Consistent with our previous observations [38] LF82 uptake was strongly reduced in the presence of 6MP (Figure 2A), suggesting that LF82 adhesion determinants may also play a role in this process. Similarly, both *Lactobacilli* and *Bifidobacteria* significantly impaired LF82 uptake by MDM derived either from HD or UC patients (Figure 2A). Surprisingly, however, no probiotic strains or 6MP, were able to affect LF82 phagocytosis by CD-derived MDM. In fact, we even observed a significant increase in the number of internalized LF82 cells from 100% to 124.9% (*p* = 0.0078) in the presence of B_2_ (Figure 2A). An even stronger enrichment was observed in coinfection experiments carried out with *S. epidermidis* on MDM derived either from UC (from 100% to 160.6%, *p* = 0.0006) or CD patients (from 100% to 236.9%, *p* = 0.0039), but not on MDM derived from HD (*p* = 0.7344).

Reduction in LF82 phagocytosis by HD-derived MDM in the presence of *L. acidophilus LA1* (L_1_), namely, the strain promoting the highest decrease in LF82 uptake in HD and UC patients (Figure 2A), was confirmed by immunofluorescence assays (Figure 2B).

We also determined LF82 survival within macrophages after 8 h of infection (Figure 2C). LF82 was unable to replicate in MDM originating from HD, where their intramacrophage concentrations remained constant during 8 h of infection, or in MDM from UC patients, where intracellular LF82 concentration was reduced to a half (Supplementary Table S2). In contrast, despite a significantly lower uptake, LF82 appeared to survive and replicate more efficiently in MDM from CD patients. Our results confirm previous reports showing that replication was not related to the initial phagocytosis level [38] and suggest that CD-derived MDM may be impaired in LF82 killing (Supplementary Table S2), consistent with deficient autophagy mechanisms observed in CD macrophages [45].

In the presence of probiotic strains, intracellular LF82 concentrations were only slightly, albeit significantly, reduced in MDM derived from either HD or UC patients, while being unaffected in CD-derived MDM, with the sole exception of a slight, but significant reduction induced by *B. animalis* spp. *lactis* Bi1 (B_1_) (Figure 2C). Interestingly, LF82 survival within UC- and CD-derived MDM was significantly higher in coinfections with *S. epidermidis* (Figure 2C), in line with an increase in LF82 uptake by IBD-derived macrophages. In contrast, a stronger reduction of LF82 survival in macrophages was observed in the presence of 6MP, regardless of MDM origin (HD, UC or CD). It is unlikely that this effect may be due to the immunosuppressive and apoptosis-promoting functions of this drug, as 2 µg/mL of 6MP was unable to induce MDM cell death as assessed by viable cell counting after 8 h of treatment (data not shown).

### 3.3. Probiotic Strains Reduced TNF-α Secretion by AIEC-Infected MDM from HD, but Not from UC or CD Patients

**Figure 3:**
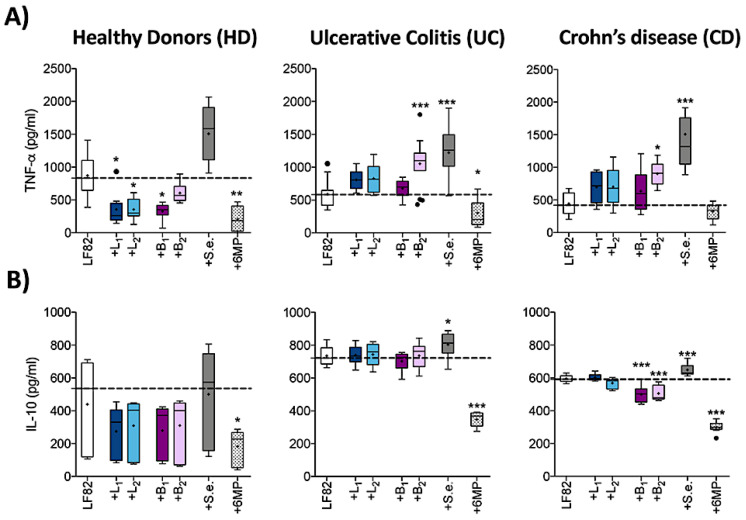
Effects of probiotic strains on cytokine secretion by MDM derived from HD, UC and CD patients infected with AIEC-LF82 strain. (**A**) TNF-α and (**B**) IL-10 secretion by MDM derived from HD, UC patients or CD patients after 8 h of infection with AIEC-LF82 alone or in the presence of *Lactobacillus* strains (+L_1_, +L_2_) or *Bifidobacterium* strains (+B_1_, +B_2_) or *S. epidermidis* ATCC-155 (+S.e.), at 1:1 ratio or in the presence of 6MP (2 µg/mL) was quantified by ELISA. Dot lines (---) represent the median values for cytokine secretion of MDM infected with LF82 alone. Data are represented as explained in Figure 1. Statistical significance for each condition compared to MDM infected with LF82 alone was reported (* *p* < 0.05, ** *p* < 0.01, *** *p* < 0.001).

In order to understand whether probiotic strains were able to modulate the inflammatory response induced by LF82 in MDM, we quantified the levels of proinflammatory cytokine TNF-α, whose levels positively correlate with LF82 intracellular survival and replication [18] and the amount of anti-inflammatory cytokine IL-10, an important regulatory cytokine which counteracts the proinflammatory effects of TNF-α (Figure 3).

Consistent with literature data [45], the TNF-α/IL-10 ratio is higher in HD-derived MDM compared to either UC or CD patients (Figure 3), thus confirming the positive correlation with these cytokine levels and LF82 intracellular amounts (Supplementary Table S2).

In HD-derived MDM, probiotic strains induced a significant reduction not only of TNF-α levels (Figure 3A), in line with the lower LF82 intracellular concentration, but also of IL-10 secretion (Figure 3B). In contrast, in UC-derived MDM, despite a significant reduction of intracellular LF82 load, TNF-α and IL-10 levels were unaffected by probiotics, except for *B. breve* Bbr8 (B_2_), which induced a significantly increase of TNF-α secretion (*p* = 0.0001). Similarly, in CD-derived MDM, in which probiotics did not affect intracellular LF82 load, TNF-α release was similar or even increased, especially in the presence of B_2_ (from 417.2 pg/mL to 900.7 pg/mL, *p* = 0.02). Surprisingly, in contrast to their proposed stimulatory effect on IL-10 secretion in macrophages [46,47] both *Bifidobacterium* strains significantly inhibited the secretion of IL-10 in MDM derived from CD patients (*p* < 0.0001).

The TNF-α increase observed in UC- or CD-derived MDM in the presence of probiotic strains was not due to their ability to promote TNF-α secretion, as MDM infection with probiotic strains alone resulted in non-detectable levels of TNF-α (Supplementary Figure S2). In contrast, coinfection with *S. epidermidis* induced a striking increase in cytokine release by MDM, regardless of their origin (*p* < 0.001), likely due to the direct induction of both TNF-α and IL-10 secretion by this bacterium (Supplementary Figure S2). Finally, exposure to the anti-inflammatory drug 6MP resulted in a significant reduction of cytokine secretion in HD- and UC-derived MDM (*p* < 0.01) and of IL-10 in CD-derived MDM, while TNF-α was unexpectedly unaffected (*p* = 0.9380).

### 3.4. Probiotic Strains Reduced AIEC-LF82 Survival and Production of Pathogenic Th17 Polarizing Cytokines in MoDC Isolated from HD and UC Patients, but Not from CD Patients

**Figure 4:**
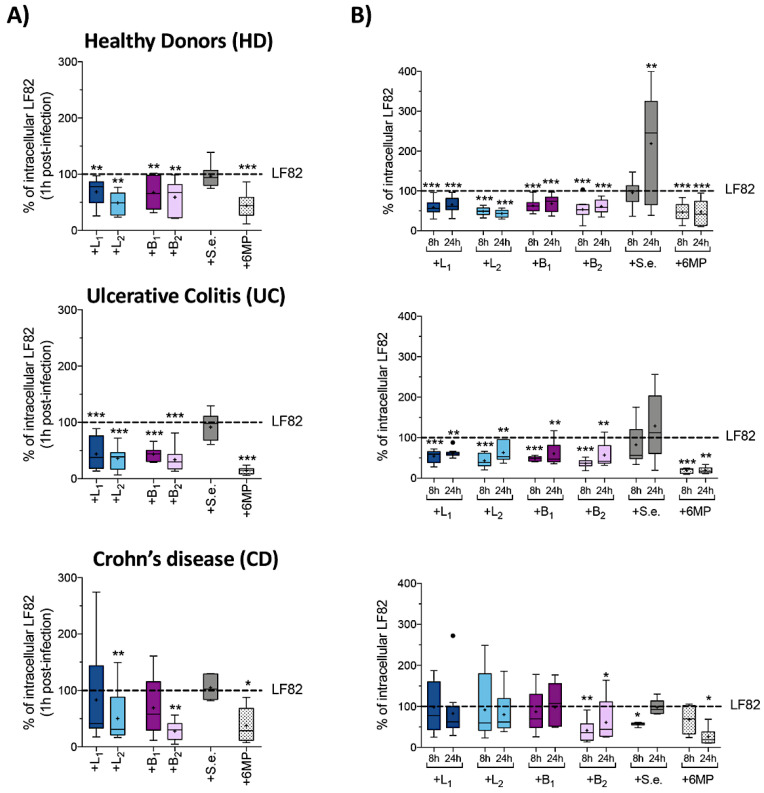
Effects of probiotic strains on phagocytosis and intracellular survival of AIEC-LF82 within human monocyte-derived dendritic cells (MoDC). Percentage of internalized AIEC-LF82 cells within MoDC derived from HD, UC or CD patients after 1 h (**A**), 8 and 24 h of infection. (**B**) MoDC were infected with LF82 alone (taken as 100%) or in the presence of *Lactobacillus* strains (+L_1_, +L_2_) or *Bifidobacterium* strains (+B_1_, +B_2_) or *S. epidermidis* ATCC-155 (+S.e.), at 1:1 ratio or in the presence of 6MP (2 µg/mL). Each experiment was performed in triplicate and data are represented as described in Figure 1. Statistical significance for each condition compared to MoDC infected with LF82 alone was reported (* *p* < 0.05, ** *p* < 0.01, *** *p* < 0.001).

The interplay between AIEC and DCs is a crucial step for the secretion of polarizing cytokines, such as IL-12p70, IL-23 and IL-1β, in turn driving Th1 or Th17 differentiation and expansion that is the most distinctive immunological characteristic of CD [19,48]. In order to establish the ability of probiotic strains to reduce AIEC uptake and survival within monocyte-derived dendritic cells (MoDC), as well as to modulate the production of proinflammatory polarizing cytokines, we coinfected MoDC with AIEC LF82 and single *Lactobacillus* or *Bifidobacterium* strains in a 1:1 ratio (Figure 4). As for the previous experiments, the effects of *S. epidermidis* and 6MP were also tested for comparative purposes.

In contrast to what observed for MDM, MoDC from HD were not significantly more efficient in LF82 uptake compared to CD patients (*p* = 0.279), while UC-derived MoDC were even less efficient in LF82 uptake compared to HD (*p* = 0.0152), suggesting that they may be somehow impaired in LF82 recognition (Supplementary Table S3).

All probiotic strains significantly reduced LF82 uptake in both HD- and UC-derived MoDC (Figure 4A). In contrast, only *L. paracasei 101/37* (L_2_) and *B. breve* Bbr8 (B_2_) strains, despite not being able to affect LF82 uptake by MDM, were able to significantly reduce the number of phagocytized LF82 (−68.9% *p* = 0.0068 and −70% *p* = 0.0010; respectively) in MoDC derived from CD patients.

Finally, in line with what observed with HT29 and MDM cells, 6MP strongly inhibited the uptake of LF82 in MoDC derived either from HD or from IBD patients (Figure 4A), while coinfection with *S. epidermidis* did not affect LF82 uptake by MoDC, regardless of their origin, further confirming that a nonprobiotic Gram-positive bacterium cannot impair LF82 interaction with human innate immune cells.

Interestingly, LF82 was not able to replicate within MoDC regardless of their origin: indeed, intracellular LF82 cells slumped, over 24 h, to 25%–40% of the number initially taken up (Supplementary Table S3). In HD- and UC-derived MoDC both *Lactobacillus* and *Bifidobacterium* strains were able to maintain a significantly lower number of intracellular LF82 cells over 24 h post-infection (Figure 4B). In contrast, in CD-derived MoDC, although L_2_ and B_2_ strains were able to strongly interfere with LF82 uptake (Figure 4A), they failed to maintain a lower intracellular LF82 bacterial load (Figure 4B), as we observed an increased percentage of LF82 within MoDC from 8 h to 24 h post-infection (from −68.9% to −37.7% with L_2_, and from −70% to −38.7% with B_2_, respectively). Coinfection with *S. epidermidis*, as well as exposure to 6MP, did not substantially alter the kinetics of LF82 killing over 8 h and 24 h (Figure 4B).

**Figure 5:**
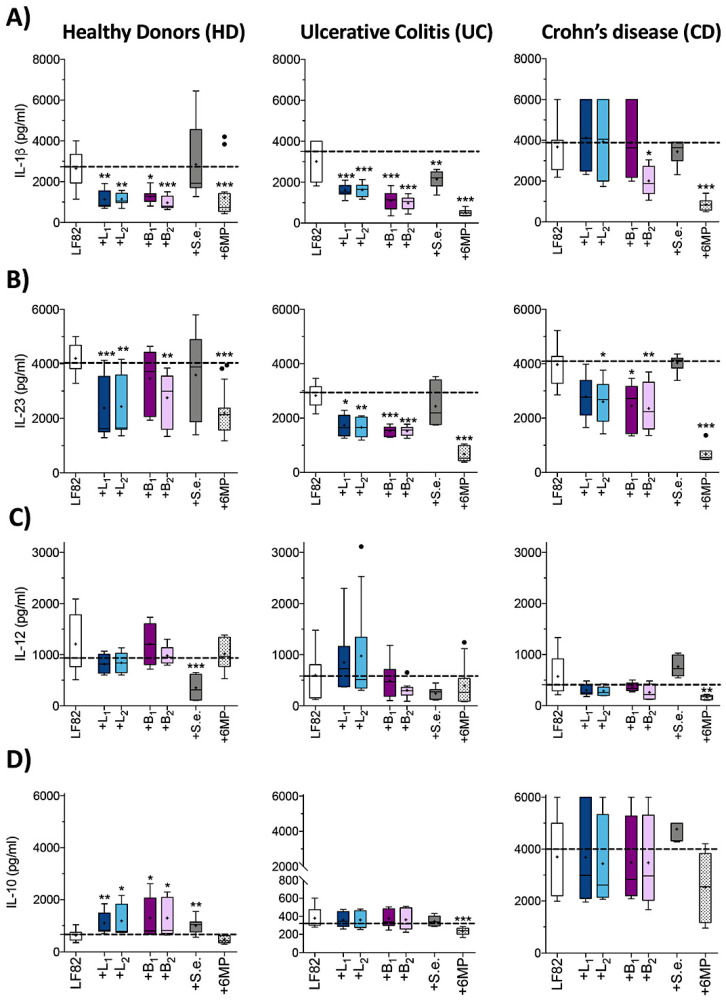
Effects of probiotic strains on cytokine secretion by MoDC derived from HD, UC and CD patients infected with AIEC-LF82 strain. Secretion of IL-1β (**A**), IL-23 (**B**), IL-12 (**C**) and IL-10 (**D**) in the supernatants of MoDC derived from HD, UC patients or CD patients after 24 h of infection with AIEC-LF82 alone or in the presence of either *Lactobacillus* strains (+L_1_ or +L_2_) or *Bifidobacterium* strains (+B_1_ or +B_2_) or *S. epidermidis* ATCC-155 (+S.e.), at 1:1 ratio or in the presence of 6MP (2 µg/mL), was quantified by ELISA. Dot lines (---) represent the median values for cytokine secretion of MoDC infected with LF82 alone. Data are represented as described in Figure 1. Statistical significance for each condition compared to MoDC infected with LF82 alone was reported (* *p* < 0.05, ** *p* < 0.01, *** *p* < 0.001).

As dendritic cells can produce both proinflammatory (IL-1β, IL-23 and IL-12) and anti-inflammatory (IL-10) cytokines involved in differentiation and regulation of adaptive immune response, we investigated whether the probiotic strains could alter the pattern of cytokine secretion by LF82-infected MoDC. Interestingly, probiotics affected cytokine release in a manner clearly dependent on the origin of MoDC (Figure 5). Indeed, LF82 coinfection with either *Lactobacilli* or *Bifidobacteria* strains in HD-derived MoDC resulted in the inhibition of the proinflammatory polarizing cytokines IL-23 and IL-1β, while stimulating IL-10 secretion, with only IL-12 levels remaining unaffected. Surprisingly, *S. epidermidis* was also able to promote IL-10 release, and, unlike any probiotic strain tested, to impair IL-12 secretion (Figure 5C, left panel). Similarly, in UC-derived MoDC only the release of proinflammatory cytokines IL-23 and IL-1β was significantly reduced by probiotic strains, while no detectable changes in IL-10 were observed (Figure 5D, center panel). Interestingly, in CD-derived MoDC, the ratio between the amount of IL-1β, IL-23 and IL-10 and the number of intracellular LF82 cells after 24 h of infection was significantly higher compared to HD-derived MoDC (Figure 5 and Supplementary Table S3), while IL-12 levels were significantly lower (Figure 5). Moreover, in coinfection experiments with probiotic strains, although the amount of intracellular LF82 was unaffected at 24 h post-infection within CD-derived MoDC (Figure 4B), we observed a sharp decrease of IL-23 secretion (Figure 5B) especially in the presence of *B. breve* Bbr8 (B_2_, *p* = 0.0099), which, unlike the other probiotic strains, was also able to significantly reduce the levels of IL-1β (*p* = 0.03). No stimulatory effect was observed on the very high levels of the anti-inflammatory IL-10 secreted by CD-derived MoDC by any probiotic strain tested (Figure 5D).

Remarkably, when we analyzed cytokine secretion by MoDC (from HD, UC and CD) only infected with single probiotic strains alone, our results displayed undetectable or very low cytokine levels, except for B_2_, which induced secretion of high levels of IL-23, IL-1β and IL-10 selectively in CD-derived MoDC (Supplementary Figure S3).

Unlike probiotic strains, which were able, at least in HD-derived MoDC, to redirect LF82-dependent cytokine production, 6MP led to a nonspecific impairment of both proinflammatory and anti-inflammatory cytokine production. Indeed, although we observed a significant reduction of IL-1β and IL-23 secretion in all MoDC analyzed, 6MP also impaired IL-10 in UC-derived MoDC (Figure 5D). In addition, 6MP was able to inhibit IL-12 levels exclusively in CD-derived MoDC, thus showing a rather different patterns of cytokine inhibition in MoDC of different origins compared to probiotic strains.

## 4. Discussion

Targeting gut dysbiosis and overly intestinal inflammation with resident microbial-targeted therapies is an attractive strategy for IBD treatment [49,50].

In this work, we analyzed the ability of two *Lactobacillus* and two *Bifidobacterium* species, arguably among the most widely used probiotic strains [51], to counteract, along different steps of the mucosal immune response, the virulence mechanisms and relative inflammatory response of the *E. coli* LF82, representative of the AIEC pathovar closely related to the IBD pathogenesis [15]. We chose these probiotic bacteria since they are an important part of normal human gut microbiota; they are very well characterized biologically and are widely used in treating dysbiosis, especially for their ability to outcompete enteropathogens [52,53]. We focus on individual strain, rather than their combination, in order to single out any strain-specific contributions to inhibition of AIEC virulence and immune cell responses.

Our results confirm the proficiency of both *Lactobacillus* and *Bifidobacterium* strains analyzed in this study in inhibiting LF82 adhesion to and invasion of IECs, which represent the first line of interaction between host and pathogens in the gut mucosa. Moreover, we observed a reduced adhesion index to abiotic surface in mixed culture biofilm experiments, suggesting that these probiotic strains probably sequester or inhibit the expression of LF82 adhesion determinants rather than via a competition mechanism for host cellular receptor sites on IECs.

Interaction of IECs with pathogens or pathobiont bacteria like AIEC induce the release of IL-8 and CCL20, two proinflammatory chemokines produced at significantly higher amounts in the inflamed gut mucosa of IBD patients [54,55], thus suggesting a pivotal role of this process in induction of chronic inflammation. Interestingly, our results show that, even if *Lactobacillus* were more proficient than *Bifidobacterium* strains to counteract the persistence of LF82 strain within HT29 cells over seven hours of infection, the secretion of IL-8 was strongly suppressed by any probiotic species tested. This outcome indicates that the amount of intracellular LF82 does not correlate with IL-8 levels and suggest different molecular mechanisms behind the effects of *Lactobacillus* and *Bifidobacterium* species on AIEC persistence within IECs and on inflammation. Moreover, our results do not show any effect on CCL20 secretion by any probiotic species tested, in stark contrast with the effect of the anti-inflammatory drug 6MP, which totally abolished the release of both IL-8 and CCL20. Lack of CCL20 inhibition by *Lactobacillus* and *Bifidobacterium* species is not due to a direct induction of this cytokine by probiotic strains, suggesting that, unlike anti-inflammatory drugs such as 6MP, probiotics can act specifically on IL-8 signaling. This immunomodulatory effect of probiotic strains is of particular importance, as the CCR6–CCL20 axis is a crucial pathway in the recruitment of macrophages, DCs and in the maintaining of Th17/regulatory T cells (Treg) balance during the initiation of immune tolerance [56,57,58]. Inhibition of CCL20-mediated cell chemotaxis and antimicrobial activity may in fact enhance susceptibility to infections, further promoting the circuit of dysbiosis and chronic inflammation behind IBD pathogenesis, suggesting that blocking CCR6-CCL20 axis may not be a long-term effective therapeutic approach to IBD [59,60]. Thus, we propose, in contrast to literature data [55], that the ability to selectively inhibit IL-8, but not CCL20, secretion may be a crucial property to consider in the screening of probiotic bacteria for their immunomodulatory activity, in order to identify the most suitable probiotic strains for long-term treatment of IBD.

Another key player in intestinal inflammation during IBD is the cytokine TNF-α [61]. Indeed, very high TNF-α levels are present in the gut mucosa of IBD patients and positively correlate with clinical disease severity, thus suggesting a negative contribution for TNF-α to the chronic intestinal inflammation. In addition, AIEC bacteria are able to use TNF-α to foster their intracellular replication within macrophages [18], as they induce the release of high levels of TNF-α, which in turn promotes AIEC intramacrophage replication, in a circuit of inflammation and infection contributing to the gut inflammation and epithelial cells damage in IBD.

In the conditions used in this work (M-CSF differentiated MDM, MOI 1:10), we observed significant differences in AIEC-LF82 persistence within human macrophages derived from IBD patients compared to HD: whereas LF82 is able to survive, but not to replicate, within MDM derived from HD or UC patients, our data confirm the proficiency of LF82 to replicate within CD-derived macrophages [62]. Our results show that, although both *Lactobacillus* and *Bifidobacterium* strains could counteract LF82 phagocytosis and intracellular survival within macrophages derived from HD and UC patients, they resulted ineffective in CD-derived macrophages. In addition, probiotic strains completely failed to block TNF-α production and to promote IL-10 secretion, a key player in the control of inflammatory response to enteric microorganisms [63,64], neither in UC-derived MDM, despite a significant lower LF82 intracellular number at eight hours post-infection, nor in MDM derived from CD patients. Surprisingly, we even observed a sharp stimulation by some probiotic bacteria in TNF-α production by AIEC-infected macrophages derived either from UC or CD patients, such as for instance in the presence of *B. breve* Bbr8, similar to what observed with the nonprobiotic *S. epidermidis*, suggesting that commensal or even probiotic strains, can synergize with LF82 in triggering proinflammatory cytokine release in CD. Thus, our data indicate that the tested probiotic strains can impair the uptake and persistence of LF82 within macrophages derived from HD and UC patients, but their immunomodulatory effects only take place on HD-derived macrophages. Indeed, CD-derived macrophages result completely refractory to the beneficial effects of probiotic strains, explaining -at least in part- the negative results yielded in some clinical trials on the use of *Lactobacillus* and *Bifidobacterium* strains in CD patients [37]. This observation, together with clinical evidences that up to 30% of IBD patients do not respond to anti-TNF-α therapies [65,66,67], underlines the urgent need to identify more promising probiotic strains which could be able to interfere with the TNF-α pathway even in IBD patients.

In contrast to the negative effect observed in AIEC-infected macrophages, *B. breve* Bbr8 strain leads to a strong reduction in AIEC intracellular persistence and proinflammatory response in IBD-derived MoDC, thus underlining the complexity of probiotics’ immunomodulatory activity in immune cells from IBD patients.

Interaction of probiotic bacteria with DCs does not only lead to a downregulation of proinflammatory polarizing cytokines, promoting the tolerogenic activity of DCs by increasing production of anti-inflammatory cytokine IL-10 [68,69], but it is also crucial in preventing the onset of chronic inflammation. Indeed, in addition to their fundamental roles in maintaining tolerance and immune homeostasis in the gut, DCs accumulate at sites of intestinal inflammation inducing the activation of bacteria-specific T cell subsets present in the lamina propria, finally contributing to the IBD pathology [70,71]. Furthermore, with growing data supporting the pathogenic role of the IL-23/Th17 axis in IBD [31,72] and with DCs being the main source of IL-23, it is becoming evident that the suppression of the microbial determinants triggering this deleterious inflammatory process in DCs, while restoring the eubiotic composition of the gut microbiota, may be an attractive therapeutic strategy in IBD [73].

Even if LF82 does not replicate within DCs and the intracellular number of LF82 is significantly lower in MoDC derived from CD patients compared to HD, CD-derived MoDC secrete a much higher amount of both proinflammatory and anti-inflammatory cytokines. This suggests a dysregulated cytokine production in CD-derived MoDC in response to bacterial antigens, in what is likely a crucial step in establishing chronic inflammation typical of this disease.

Notably, our data demonstrate for the first time that LF82 can induce a different release of polarizing cytokines, which lead to distinct effector Th-cell subsets polarization, on the basis of DCs origin: under the same intracellular amount of LF82 cells after 24 h of infection, CD-derived MoDC secrete higher amount IL-23 compared to UC-derived MoDC promoting expansion of pathogenic Th17 cells, while, on the contrary, UC-derived MoDC secrete significantly higher amount of IL-12 driving instead the differentiation into Th1 cells. The molecular mechanisms by which AIEC-infected DCs induce secretion of distinct patterns of polarizing cytokines, in turn leading to opposite differentiation of effector T cells associated with the different immunophenotypes in UC or CD patients, is probably due to many genetic variants/polymorphisms that could directly affect the function of professional phagocytic cells, as suggested by several independent studies [45,62,74].

Altogether, our data demonstrate that in HD and UC patients the *Lactobacillus* and *Bifidobacterium* strains studied in this work interfere with the uptake and persistence of LF82 within dendritic cells, and are all able to interfere with the IL-23/Th17 axis, to a similar extent as the anti-inflammatory drug 6MP, while also stimulating production of the anti-inflammatory cytokine IL-10. In contrast, in CD-derived dendritic cells, probiotic strains, except for *B. breve* Bbr8 strain, are much less effective in affecting LF82-induced inflammatory response and in hampering release of the polarizing IL-1β and IL-23 cytokines that regulate differentiation of pathogenic Th17 cells [75,76]. Moreover, the protective effect of probiotic strains on DCs, in addition to downregulating proinflammatory cytokines, should promote tolerance-inducing DCs through the upregulation of IL-10, which plays an important regulatory role on effector T-cells [77]. In this respect, the different effects displayed by both *Lactobacillus* and *Bifidobacterium* strains in MoDC derived from HD vs. IBD patients further highlight the importance to test the immunoregulatory activities of probiotics on activated immune cells derived from IBD patients, in order to fully evaluate their therapeutic potential.

Importantly, the differential impact of probiotic strains on AIEC virulence and inflammatory response in IBD-derived immune cells may also have important clinical implication. Indeed, in contrast to UC patients, results of clinical trials in the treatment of active CD with probiotics are disappointing and do not support their use in this disease [78,79,80]. However, the observation that only some probiotic bacteria, such as the *B. breve Bbr8* strain, displayed the ability to affect some inflammation pathways also in CD patients, such as the secretion of polarizing cytokines involved in the IL-23/Th17 axis, underline the importance of testing probiotic strains in IBD-derived immune cells in order to select the most suitable strains that may find some therapeutic use also in CD.

Finally, a deeper molecular analysis aimed at characterizing the interactions of probiotics with immune cell signaling cascades will be crucial in identifying probiotic strains able to inhibit the activation of IL-23/Th17 axis, without increasing of TNF-α or inhibition of IL-10 as potential side effects, likely to be a more effective therapeutic strategy for UC and CD.

## Figures and Tables

**Table 1:** Bacterial strains, origin and relevant characteristics used in this study.

Strains	(BioProject Number) and Reference	Relevant Characteristic
B_1_—*Bifidobacterium animalis* spp. *Lactis Bi1*	(n/a) ^§^ this work ^#^	Probiotic strain
B_2_—*Bifidobacterium breve Bbr8*	(n/a) ^§^ this work ^#^	Probiotic strain
L_1_—*Lactobacillus acidophilus LA1*	(PRJNA340059) this work ^#^	Probiotic strain
L_2_—*Lactobacillus paracasei 101/37*	(n/a) ^§^ this work ^#^	Probiotic strain
LF82—*Adherent-Invasive Escherichia coli* (AIEC)	(PRJNA487828) [38]	Crohn’s disease (CD)-associated *E. coli* strain
S.e.—*Staphylococcus epidermidis* ATCC-155	(n/a) ^§^ [39]	Laboratory nonprobiotic strain

^§^ n/a—not available; ^#^—provided by Named^®^ SpA (Milan, Italy).

## Supplementary Materials

The following are available online at https://www.mdpi.com/2073-4409/9/8/1824/s1, Figure S1: Biofilm formation in the presence of probiotic strains and cytokine secretion by HT29 cells infected with probiotic strains alone. Figure S2: Cytokine secretion by MDM infected with probiotic strains alone. Figure S3: Cytokine secretion by MoDC infected with probiotic strains alone. Table S1: Clinical characteristics of Crohn’s disease (CD), ulcerative colitis (UC) patients and healthy subjects (HD) included in the study. Table S2: Phagocytosis and survival of AIEC-LF82 within MDM obtained from HD, UC and CD patients. Table S3: Phagocytosis and survival of AIEC-LF82 within MoDC derived from HD, UC and CD patients.
