# Supplementary material for: Probiotic Lactobacillus and Bifidobacterium Strains Counteract Adherent-Invasive Escherichia coli (AIEC) Virulence and Hamper IL-23/Th17 Axis in Ulcerative Colitis, but Not in Crohn’s Disease
Source: Cells. 2020 Aug 1;9(8):1824. doi: 10.3390/cells9081824 (PMC7464949; doi:10.3390/cells9081824)
Supplement: Supplementary file 1 [file cells-09-01824-s001.pdf]

Figure S1

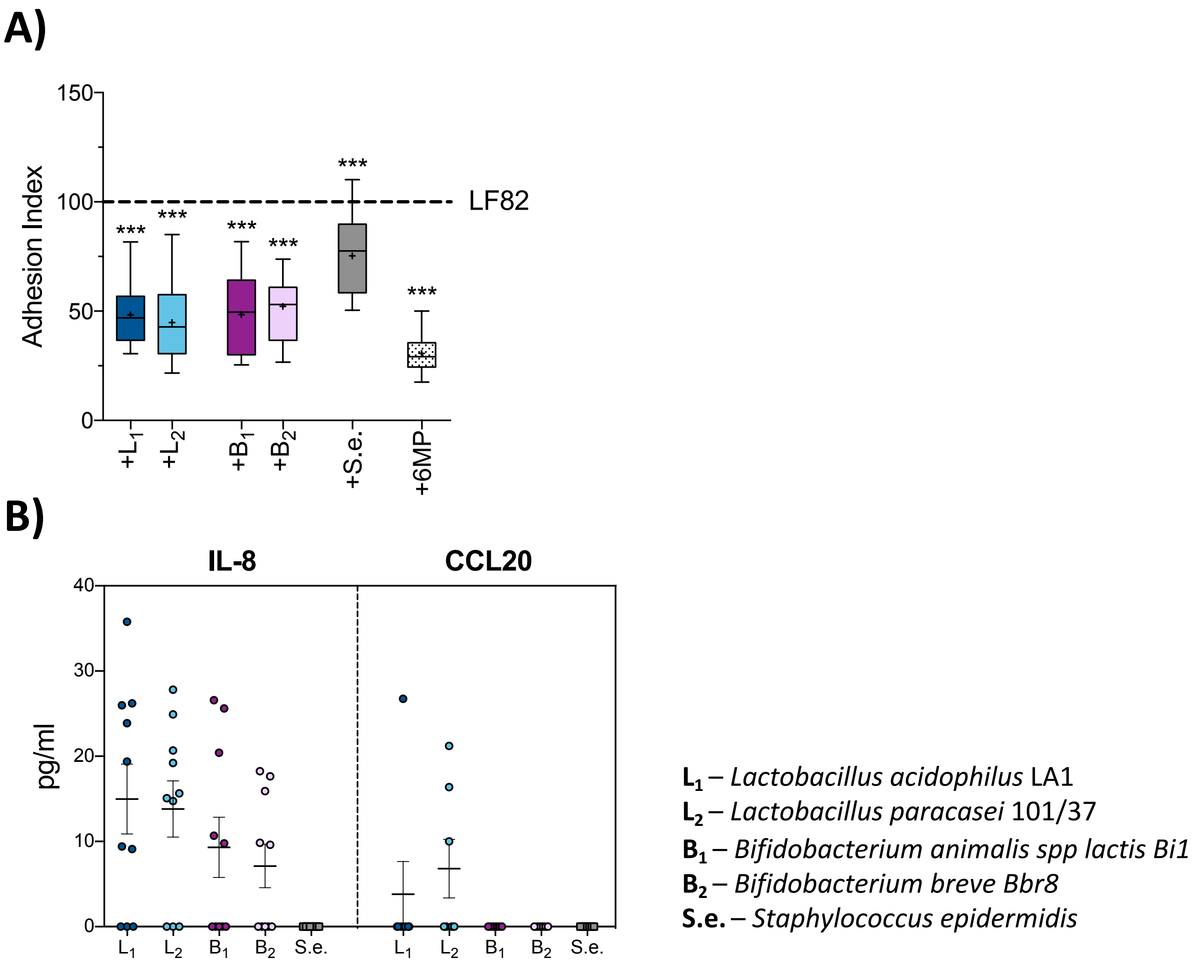

Figure S1: **Biofilm formation in the presence of probiotic strains and cytokine secretion by HT29 cells infected with probiotic strains alone.**

(A) Adhesion to abiotic surface of AIEC-LF82 alone (taken as 100%), or in the presence of *Lactobacillus acidophilus* LA1 (+L_1), *Lactobacillus paracasei* 101/37 (+L_2), *Bifidobacterium animalis spp lactis* Bi1 (+B_1), *Bifidobacterium breve* Bbr8 (+B_2), *S. epidermidis* ATCC-155 (+S.e.) at 1:1 ratio, or in the presence of 6-mercaptopurine (+6MP, 2 μg/ml).
Data are represented with Box-plots showing the median, range and upper and lower quartiles of at least three independent experiments. Means are represented as black cross (+), median as horizontal lines, and outliers are marked as dots. Statistical significance for each condition compared to AIEC-LF82 alone (taken as 100%) was reported (*p<0.05, **p<0.01, ***p<0.001).

(B) IL-8 and CCL20 secretion by HT29 cell monolayers infected with *Lactobacillus* strains (L_1, L_2), or *Bifidobacterium* strains (B_1, B_2), or *S. epidermidis* ATCC-155 (S.e.) was quantified by ELISA. Data are represented as mean $\pm$ SEM of at least three independent experiments.

Figure S2

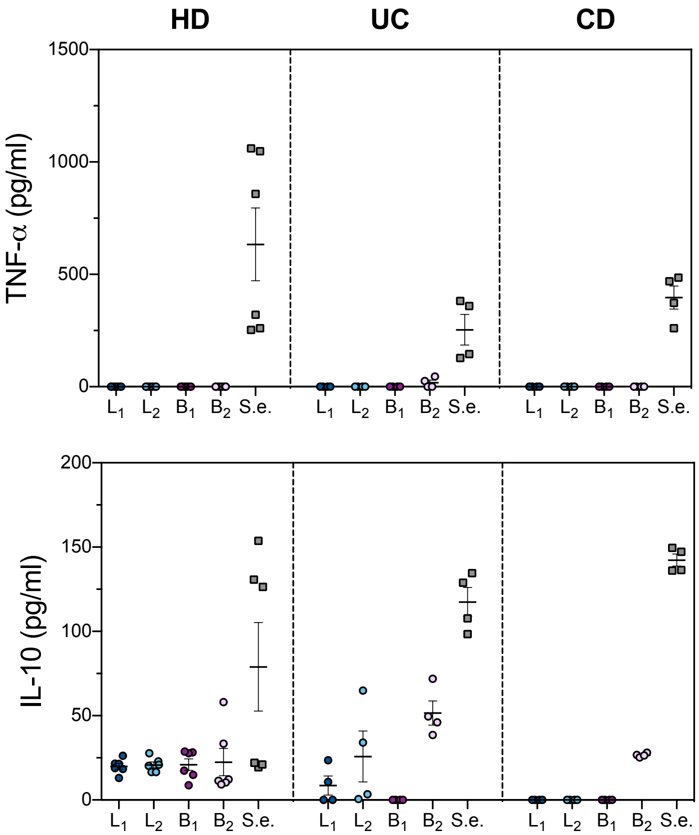

L_1 – *Lactobacillus acidophilus* LA1
L_2 – *Lactobacillus paracasei* 101/37
B_1 – *Bifidobacterium animalis spp lactis Bi1*
B_2 – *Bifidobacterium breve Bbr8*
S.e. – *Staphylococcus epidermidis*

Figure S2: Cytokine secretion by MDM infected with probiotic strains alone.

TNF-α and IL-10 secretion by MDM derived from HD, UC or CD patients infected with *Lactobacillus* strains (L_1, L_2), or *Bifidobacterium* strains (B_1, B_2), or *S. epidermidis* ATCC-155 (S.e.) was quantified by ELISA. Data are represented as mean $\pm$ SEM of at least three independent experiments.

Figure S3

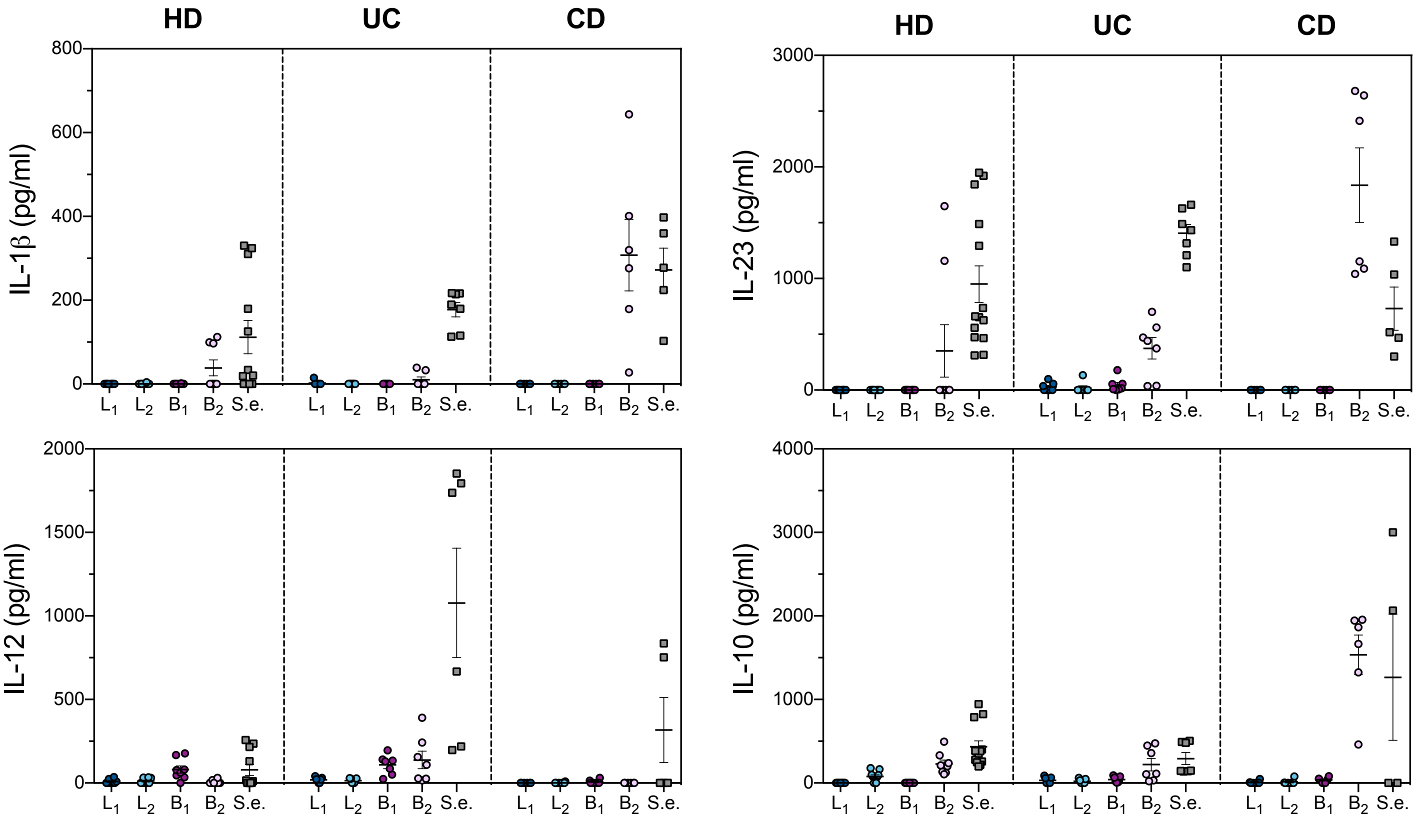

L_1 – *Lactobacillus acidophilus* LA1

L_2 – *Lactobacillus paracasei* 101/37

B_1 – *Bifidobacterium animalis spp lactis Bi1*

B_2 – *Bifidobacterium breve Bbr8*

S.e. – *Staphylococcus epidermidis*

Figure S3: Cytokine secretion by MoDC infected with probiotic strains alone.

Secretion of IL-1β, IL-23, IL-12 and IL-10 by MoDC derived from HD, UC or CD patients after 24 hours of infection was quantified by ELISA. MoDC were infected with *Lactobacillus* strains (L_1, L_2), or *Bifidobacterium* strains (B_1, B_2), or *S. epidermidis* ATCC-155 (S.e.) alone.
Data are represented as mean ± SEM of at least three independent experiments.

Table S1: Clinical characteristics of Crohn's disease (CD), Ulcerative colitis (UC) patients and healthy subjects (HD) included in the study.

CHARACTERISTICS	Healthy Donors (HD)	Ulcerative colitis (UC)	Crohn's disease (CD)
**- Male n (%)**<br>**- Female n (%)**	- 4 (40 %)<br>- 6 (60 %)	- 7 (63.6 %)<br>- 4 (36.4 %)	- 4 (50 %)<br>- 4 (50 %)
**Age year (mean±SEM)**	46.40 ± 3.51	51.36 ± 5.02	48.22 ± 5.41
**Mean age at diagnosis (mean±SEM)**	-	35.82 ± 4.74	28.89 ± 5.67
**Mean duration of disease (mean±SEM)**	-	15.64 ± 3.66	20 ± 3.70
**Former smokers (n, %)**	4 (40%)	2 (18.18%)	0 (0%)
**Previous surgery n (%)**	-	0 (0%)	6 (75%)
**Medical treatment**<br>**- none (n, %)**<br>**- 5ASA (n, %)**<br>**- steroids (n, %)**<br>**- antibiotics (n, %)**	-	 - 1 (9.09%)<br>- 8 (72.72%)<br>- 2 (18.18%)<br>- 0 (0%)	 - 2 (9.09%)<br>- 6 (72.72%)<br>- 0 (0%)<br>- 0 (0%)

Table S2: Phagocytosis and survival of AIEC-LF82 within MDM obtained from HD, UC and CD patients.

LF82 (CFU/ml)	**HD** (mean ± SEM) x10^3	**UC** (mean ± SEM) x10^3	**CD** (mean ± SEM) x10^3
**1h (phagocytosis)**	208.1 ± 19.28	197.4 ± 20.72	82.7 ± 7.21<br>(** p=0.0011 vs HD)<br>(** p=0.0012 vs UC)
**8h post-infection**	175 ± 2.48	112.4 ± 16.84<br>(** p=0.0071 vs HD)	94.53 ± 8.49<br>(** p=0.0094 vs HD)

The numbers of LF82 cells internalized within MDM were determined after 1h (phagocytosis) or after 8h of infection, and expressed as numbers of CFU/ml (mean ± SEM). Data were analyzed by One-way Anova (Dunnett's multiple comparisons):
*p<0.05, **p<0.01, ***p<0.001.

Table S3: Phagocytosis and survival of AIEC-LF82 within MoDC derived from HD, UC and CD patients.

LF82 (CFU/ml)	**HD** (mean ± SEM) x10^3	**UC** (mean ± SEM) x10^3	**CD** (mean ± SEM) x10^3
**1h (phagocytosis)**	436.9 ± 48.1	228.4 ± 20.7 (* p=0.0152 vs HD)	300.5 ± 47.9
**8h post-infection**	391.5 ± 39.8	219.1 ± 29.7 (** p=0.0325 vs HD)	141.5 ± 28.4 (*** p<0.0001 vs HD)
**24h post-infection**	144.8 ± 11.6	92.3 ± 14.7	72.51 ± 19.3 (** p=0.0025 vs HD)

The numbers of LF82 cells internalized within MoDC were determined after 1h (phagocytosis), 8h and after 24h of infection, and expressed as numbers of CFU/ml (mean ± SEM). Data were analyzed by One-way Anova (Dunnett's multiple comparisons):
*p<0.05, **p<0.01, ***p<0.001.
